# Supplementary material for: Promotion of Mental Health Literacy in Adolescents: A Scoping Review
Source: Int J Environ Res Public Health. 2021 Sep 9;18(18):9500. doi: 10.3390/ijerph18189500 (PMC8470967; doi:10.3390/ijerph18189500)
Supplement: Supplementary file 1 [file ijerph-18-09500-s001.zip › Table S1.pdf]

Table S1. Characteristics of the articles included (n = 29).

Author(s), Year	Country	Participants	Type of study	Programme / Interventions	Duration	Data collection instruments	Main outcomes	Barriers / facilitators
Lubman et al (2016)	Australia	Adolescents aged 14-15 years (Year-9 students)	Study protocol: Cluster Randomized Controlled Trial	*MAKINGtheLINK* is a universal school-based intervention to facilitate help-seeking for substance use and mental health problems, by improving the mental health literacy of young people.<br>Education on recognition when a friend needs help (vignettes), on types of helpers available, on myths and facts about substance use and mental health, on identifying and overcoming barriers to professional help-seeking, assisting a friend to access help and on accessing reliable sources of help.<br>Activities consists in provide information and also videos for skill rehearsal during the classroom.<br>It was delivered by an experienced external facilitator with the assistance of the regular classroom teacher.	5 interactive classroom activities run over 2 school periods (average period is 75 minutes), plus a booster session 1 month later	All students will complete a self-report questionnaire at baseline, immediately post intervention and 6- and 12-months post baseline:<br>- Demographic information<br>- Depression Anxiety Stress Scales (DASS-21)<br>- Australian Secondary School Students Alcohol and Drug (ASSAD) Survey<br>- General Help Seeking Questionnaire (GSHQ-V)<br>- Actual Help Seeking Questionnaire (AHSQ)<br>- 5-point Likert scale to measure the Confidence to seek help and the Confidence to seek help for a peer<br>- Barriers to Adolescents Seeking Help questionnaire (BASH-B)	Not available	Not available
Yang et al (2018)	USA	A cohort of 7th and 8th grade at-risk students (n = 14)	Pilot study: Quasi-experimental	*Integrated Science Education Outreach (InSciEd Out)* is a school-based anti-stigma intervention in mental health to improve attitudes and downstream behaviors toward mental illness through addressing mental health literacy.<br>It is an education curriculum in mental health: biogenetic explanations of mental illness, his social and cultural context; elaboration of mental health research projects; data analysis; and creation of mental health promotion art in the science, math and language arts classes.	20-day anti-stigma classroom experience	Student surveys were administered pre–post intervention:<br>- Westbrook Mental Health Knowledge Test (WMHKT)<br>- Adolescent Attribution Questionnaire (AQ-8-C)<br>- General Help-Seeking Questionnaire Vignette Version (GHSQ-V)<br>- Teacher's exit interviews	- Slight gains in mental health literacy<br>- Moderate improvements in help-seeking intentions<br>- Large decreases in mental health misconceptions<br>- Potential feasibility and acceptability of curricular-based, anti-stigma mental health interventions for at-risk youth	**Facilitators:**<br>- Students are encouraged to be active creators rather than passive consumers of knowledge

Table S1. Cont.

Author(s), Year	Country	Participants	Type of study	Programme / Interventions	Duration	Data collection instruments	Main outcomes	Barriers / facilitators
Campos et al (2014)	Portugal	Adolescents aged 12-14 years (7th, 8th and 9th grade students) (n=70)	Pilot study: - Qualitative study - Quasi-experimental	*Finding Space to Mental Health* is a school-based intervention to promote mental health literacy in young people (12-14 years old). Education on knowledge and beliefs about mental health/mental illness; on mental health problems (signs, impact, risk factors); on depression, anxiety, anorexia, schizophrenia (signs and symptoms); on non-stigmatized behaviours towards mental health disorders; on help-seeking options; on first aid skills towards people with mental health problems; and on self-help strategies. In both sessions were adopted interactive methodologies such as group dynamics, videos and music. The intervention was delivered by a trained psychologist.	2 sessions (90 minutes each), implemented with one-week interval	Student surveys were administered pre–post intervention: - Mental Health Literacy questionnaire (MHLq)	- Significant increase on knowledge, First Aid skills and help seeking, and self-help strategies on post-intervention<br>- The intervention showed itself to be adequate to promote mental health literacy in young people.	Unknown
Eschenbeck et al (2019)	Germany	Children and adolescents older than 12 years (6th to 13th grade)	Study protocol: Randomized controlled trial (RCT)	*StresSOS* is an Internet-based version of a universal school-based health promotion program that aims to improve stress management and mental health literacy in children and adolescents. Education on coping skills (problem-solving, cognitive reconstruction, relaxation techniques, seeking support); on the connection between thoughts, feelings, and behaviors, and on concepts about mental health/illness and help-seeking. Compares online intervention with face-to-face intervention: the *StresSOS* face-to-face program (classroom-based intervention), the *StresSOS* online program (from their computer at home) and the online control intervention (program for healthy nutrition).	8 weekly sessions	The surveys were administered at baseline, post intervention and some of them at 12- and 24-months post baseline: - Sociodemographic information - Psychopathology (SDQ; SEED; WCS; PHQ-A) - Alcohol misuse (CRAFFT-d; AUDIT) - Help-seeking (GHSQ; AHSQ; IATSMHS; BASH-B; MRV) - KIDSCREEN-10 - Knowledge about stress/coping, mental health (developed by authors) - Stress symptoms, coping (SSKJ 3-8R) - Self-esteem (SEKJ) - Questionnaire on Social Distance - Standardized Assessment of Personality - Abbreviated Scale (SAPAS) - Program acceptance (developed by authors)	Not available	Not available

Abbreviations: SDQ, Strengths and Difficulties Questionnaire; SEED, Short Evaluation of Eating Disorders; WCS, Weight Concerns Scale; PHQ-A, Patient Health Questionnaire-9 modified for Adolescents; CRAFFT-d, Car, Relax, Alone, Forget, Friends, Trouble questionnaire; AUDIT, Alcohol Use Disorders Identification Test; GHSQ, General Help-Seeking Questionnaire; AHSQ, Actual Help-Seeking Questionnaire; IATSMHS, Inventory of Attitudes Toward Seeking Mental Health Services; BASH-B, Barriers to Adolescents Seeking Help Scale; MRV, Mannheimer Modul zum Ressourcenverbrauch; KIDSCREEN-10, Health-related quality of life; SSKJ 3-8R, Stress and Coping Questionnaire for Children and Adolescents; SEKJ, Self-Esteem for Children and Adolescents.

Table S1. Cont.

Author(s), Year	Country	Participants	Type of study	Programme / Interventions	Duration	Data collection instruments	Main outcomes	Barriers / facilitators
Bella-Awusah et al (2014)	Nigeria	Students in junior secondary school 2 (Year 8) and senior secondary 1 (Year 10) aged between 10 and 18 years (n = 154) Intervention Group: n = 78 Control Group: n = 76	Quasi-experimental two group pre-test/post-test control group design	Mental health teaching programme is a school based mental health awareness programme aimed at increasing mental health literacy and reducing negative views about persons with mental illness.<br>Awareness and education on mental health problems (signs and behaviours), on limitations of people with mental health problems, on help-seeking options to support peers, on self-strategies to support their mental health problems and/or to maintain a good mental health.<br>It was used group discussions and brief presentations.<br>The intervention was delivered by the authors of the programme.	A single three-hour session	Students completed a questionnaire before the session started, immediately after the session and at 6 months after:<br>- A modified version of the UK Pinfold questionnaire<br>- 5 factual statements about myths and beliefs surrounding mental illness (developed by authors)<br>- 1 attitude statement (developed by authors)	- An increase in students' knowledge<br>- Slight decrease in attitude scores in post-intervention period, but returned to baseline in the follow-up<br>- Slight increase in social distance scores<br>- This programme produced significant changes only in students' knowledge but not in attitudes and social distance.<br>- Feasibility of the programme in the students	**Barriers:**<br>- The duration of the training which was very short<br>- The use of the English as language of delivery of the sessions (not the native language)
Lindow et al (2020)	USA	Students from 11 schools: 7th, 8th, 9th, 10th, 11th and 12th grades (n = 1,878 in 78 total classes)	An uncontrolled, pre-test/post-test design	*The Youth Aware of Mental Health (YAM)* intervention is a universal, school-based mental health promotion and suicide primary prevention intervention for adolescents.<br>Awareness of mental health about suicide risk and protective factors (depression, anxiety and social support). Education on skills, knowledge and emotion awareness to face stressful life events. Key themes: awareness of mental health, self-help advice, stress and crisis, depression and suicidal thoughts, helping a troubled friend, and information about mental health resources/help seeking.<br>It was used 3 role-play sessions, 2 mental health interactive lectures, an information booklet and 6 posters.	Five 50-minute sessions over the course of 3 or 5 weeks	Students completed surveys before and 3 months post intervention:<br>- Four questions on help seeking behaviors related to depression and suicidal ideation were adapted from the ongoing RCT of YAM being conducted by the YAM originators in Sweden<br>- General Help Seeking Questionnaire (GHSQ)<br>- Two mental health literacy scales, with 17 total items, were adapted from a randomized controlled trial of YAM currently being conducted in Stockholm Sweden<br>- The first four items from the Reported and Intended Behavior Scale<br>- Seven questions based on a vignette depicting an adolescent, "John," experiencing depression with suicidal ideation	- Significantly increase of mental help seeking behaviors<br>- Improve of mental health literacy<br>- Decrease of mental health-related stigma<br>- It did not affect help-seeking intent<br>- YAM is a promising mental health promotion intervention	**Facilitators:**<br>- The use of non-school personnel (certified facilitators and helpers) to deliver de YAM, promoted an openly discuss with students without fear or shame.

Table S1. Cont.

Author(s), Year	Country	Participants	Type of study	Programme / Interventions	Duration	Data collection instruments	Main outcomes	Barriers / facilitators
Hui et al (2019)	China	Adolescents aged 12-17 years (7th -12th grade students) (n = 4520)	Quasi experimental pre-test/post-test	*The School Tour* is an interventional programme, developed by the Early Psychosis Foundation (EPISO), to direct young people's attention to the important issue of stigma towards psychosis.<br>Awareness and education on psychosis (symptoms, prognosis and treatment) and self-help strategies to improve student's concentration/interest in learning and also to reduce psychotic symptoms.<br>It was used a drama performance (by professional actors), a presentation/talk and an exercise demonstration (FITMIND dance and yoga gestures)	1hour	Students completed a questionnaire before and after the session:<br>- Knowledge about Schizophrenia Test (KAST)<br>- 8 attitude statements towards the illness (developed by authors)	- Improve of knowledge about psychosis (mental health literacy)<br>- Improve of attitude towards psychosis (stigma)<br>- Effectiveness of The School Tour programme	Facilitators:<br>- The use of drama performance about true patient's stories instead of real contact with psychotic individuals<br>- The inclusion of a quiz after the presentation<br>- The incorporation of exercises and yoga
Perry et al (2014)	Australia	Secondary school students in Year 9 or 10 (aged 13-16 years) (n = 380)	Cluster Randomized Controlled Trial	*HeadStrong* is a universal, curriculum-based educational program.<br>Education on mental health and wellbeing concepts, values, perceptions, the dynamic nature of mental health, stigma, mood disorders, help-seeking, how to support a friend/colleague if he/she is experiencing mental health difficulties, self-strategies to boost mental health and prevent mental health problems (build resilience and exercise the mind) and propose/develop/implement local actions to raise awareness and dispel myths toward adolescent's mental health issues.<br>It was used a booklet, slideshow and activities implemented in the classroom by the teacher (they participated in a program training previously).	10 hours of class time in total, for 5-8 weeks	Students were assessed pre- and post-intervention, and at 6-month follow-up:<br>- Depression Literacy Scale (D-Lit)<br>- Depression Stigma Scale (DSS)<br>- Inventory of Attitudes towards Seeking Mental Health Services (IASMHS)<br>- Depression Anxiety and Stress Scales (DASS-21)<br>- Moods and Feelings Questionnaire (MFQ)	- Improve of mental health literacy<br>- Reduce of stigma toward depression<br>- It did not have impact on attitudes towards help-seeking neither on psychological distress or suicidal ideation<br>- *HeadStrong* is an effective program in improve mental health literacy and reduce stigma	Unknown

Table S1. Cont.

Author(s), Year	Country	Participants	Type of study	Programme / Interventions	Duration	Data collection instruments	Main outcomes	Barriers / facilitators
Casañas et al (2018)	Spain	Adolescents aged 11-18 years (secondary school students)	Study protocol: Cluster Randomized Controlled Trial	*EspaiJove.net* (a space for mental health) is a universal school based-intervention programme to promote mental health, prevent mental disorders, facilitate help-seeking behaviours and eradicate related stigma.<br>Education on mental health wellbeing, help-seeking behaviours, consequences of risk behaviours, mental health disorders, prevention and early detection of mental health-related problems, and on mental health-related stigma.<br>It will be use taught classes, training activities (website www.espaijove.net and online consultation) and contact with a person who had experienced mental illness firs-hand.<br>The programme will be delivered by trained mental health nurses through workshops run in the classrooms.	14 hours in total (1 hour/ week) total of 6 modules	Students will be assessed at baseline, and at 2 weeks, 6- and 12-months after the intervention:<br>- Socio-demographic information<br>- EspaiJove Mental Health Literacy Test (EMHLT) (developed by authors)<br>- Reported and Intended Behaviour Scale (RIBS)<br>- The Scaling Community Attitudes toward the Mentally Ill (CAMI)<br>- Short questionnaire to evaluate the acceptability and satisfaction of interventions (developed by authors)<br>- Strengths and Difficulties Scale (SDQ)<br>- States of Change Scale (SCS)<br>- Four yes/no questions to evaluate Bullying and cyber-bullying behaviours (developed by authors)<br>- The EuroQol 5D-5 L questionnaire (EQ-5D-5 L)<br>- Spanish version of the General Help-Seeking Questionnaire (GHSQ)<br>- Health Benefits Questionnaire	Not available	Not available
Ojio et al (2020)	Japan	Adolescents aged 15-18 years (10th -12th grades - High School students)	Descriptive paper	*The new Course of Study* is a school-based mental health education programme to promote the acquiring of the correct knowledge about mental illnesses and the utilization of the knowledge.<br>Education on mental illness (mechanism, prevalence, onset age, risk factors, symptoms, treatment), self-help strategies to prevent/ recover from mental illness, help seeking, helping behaviour and mental health-related stigma.<br>It will be use animated films, videos with filmed social contact with adolescents with mental health problems, and educators' manuals.<br>Will be delivered by teachers of health and physical education.	Unknown.<br><br>Will be effective from 2022.	Unknown	Not available	Not available

Table S1. Cont.

Author(s), Year	Country	Participants	Type of study	Programme / Interventions	Duration	Data collection instruments	Main outcomes	Barriers / facilitators
Kutcher, Bagnell & Wei (2015)	Canada	High School Students and teachers	Descriptive paper	Two complementary mental health literacy approaches:<br>1) *The Guide (The Mental Health and High School Curriculum)* is a resource to support curricula across Canada.<br>Education on stigma of mental illness, mental health and wellness, specific mental illnesses, experiences of mental illness, seeking help and finding support, and on the importance of positive mental health.<br>It is use animated videos, first voice videos, digital story-telling videos, PowerPoint® slides, in-class handouts, and Web-linked resources.<br>The intervention is delivered by the students' usual teachers.<br>2) *MyHealth Magazine* is a resource that provides online interactive health and mental health programming and materials for students and educators to increase mental health literacy, improve coping strategies, and facilitate help seeking in young people.<br>Information about health and mental health updated every week. Includes questions and answers (with topic search feature), pop-up quizzes (eg, stress quiz), and how-to sheets (eg, how to talk to your parent about a problem).<br>Was created a phone application version.	Between 8 and 12 hours (Total duration of in-class intervention – 6 modules)	Unknown	Based on 2 quasi-experimental studies and 1 RCT:<br>*The Guide*:<br>- Improve of students and teacher's mental health literacy<br>- Improve of self-reported help-seeking<br><br>*MyHealth Magazine*:<br>- High percentage of student's access<br>- Might satisfaction rating of Website from students and schools	**Facilitators:**<br>- This approach builds on existing school ecologies with 3 common elements: teachers, students, and curriculum
Mcluckie et al (2014)	Canada	Students in 9th grade in high schools (n=265)	Secondary cross-sectional survey analysis	*The Guide* (The Mental Health and High School Curriculum) is a resource to support curricula across Canada.<br>Education on stigma of mental illness, mental health and wellness, specific mental illnesses, experiences of mental illness, seeking help and finding support, and on the importance of positive mental health.<br>It was used didactic instruction, group discussion, group activities, self-directed learning and video presentations.<br>The intervention was delivered by the students' usual teachers.	Total of 10/12 hours of class time	Students were assessed pre- and post-intervention, and at 2-month follow-up:<br>- Knowledge and attitude survey (developed by authors)	- High improvement in knowledge<br>- Decrease in stigmatizing attitudes toward mental disorders/illness	**Facilitators:**<br>- Such an approach does not require significant amounts of external resources added to schools and is not dependent upon mental health experts

Table S1. Cont.

Author(s), Year	Country	Participants	Type of study	Programme / Interventions	Duration	Data collection instruments	Main outcomes	Barriers / facilitators
Gonçalves et al (2016)	Portugal	Adolescents in 7th - 9th grade (n = 207) Intervention Group: n = 115 Control Group: n = 92	Pilot study: Randomized Controlled Trial	*Program for the de-stigmatization of youth mental health* is a video-based intervention. A video administered intervention to reduce stigma related to mental health problems. It was used a video about an adolescent who is experiencing a common mental health problem (tobacco and substance use), in which she describes her problem and barriers, and the positive and the negative aspects of mental health care.	A single 10-minute session	Students were assessed pre- and post-intervention, and at 1-month follow-up: - Self Stigma of Seeking Help Scale (SSOSH) - Social Stigma for Receiving Psychological Help Scale (SSRPH) - Attribution Questionnaire-Children form (AQ-8c)	- Decrease in mental health-related stigma - This program is a promising quick and inexpensive intervention with short-term impact on adolescents' attitudes towards peers with mental health problems	Unknown
Santos et al (2013)	Portugal	Adolescents in the 3rd and secondary cycle (7th – 12th grade)	Descriptive paper	*Projeto +Contigo* is a multilevel network intervention that aims promote mental health and wellbeing in adolescents, prevent suicidal behavior, combat stigma in mental health, and create a mental health care network. Education on stigma, adolescence, self-esteem, problem-solving skills, wellbeing, assertive communication, management of emotions and risky factors. It is use presentations, group discussions, role-play and socio-therapeutic games.	Six 45-minute sessions	Students are assessed pre- and post-intervention, and at 6-month follow-up: - Questionnaire with Socio-demographic information and scales to characterize wellbeing, coping, depression and self-concept	Not available	**Facilitators:** - Involves the existing structures/ resources, creating synergies at the community level
Campos et al (2018)	Portugal	Adolescents aged 12-14 years (7th, 8th and 9th grade students) (n = 543) Intervention Group: n = 259 Control Group: n =2 84	Randomized Controlled Trial	*Finding Space to Mental Health* is a school-based intervention program focused on the promotion of mental health literacy in young people. Education on knowledge and beliefs about mental health/mental illness; on mental health problems (signs, impact, risk factors); on depression, anxiety, anorexia, schizophrenia (signs and symptoms); on non-stigmatized behaviours towards mental health disorders; on help-seeking options; on first aid skills towards people with mental health problems; and on self-help strategies. In both sessions were adopted interactive methodologies such as group dynamics, videos and music. The intervention was delivered by a trained psychologist.	2 sessions (90 minutes each), implemented with one-week interval	Students are assessed pre- and post-intervention, and at 6-month follow-up: - Mental Health Literacy questionnaire (MHLq)	- Increase on knowledge - Decrease on stereotypes - Increase on first aid Skills and help-seeking - Increase on self-help strategies - Showed to be effective on promoting mental health literacy in young people. - Showed the usefulness of short interventions to increase knowledge about mental health problems in young people	Unknown

Table S1. Cont.

Author(s), Year	Country	Participants	Type of study	Programme / Interventions	Duration	Data collection instruments	Main outcomes	Barriers / facilitators
Mellor (2014)	UK	Children or adolescents attending primary or secondary school (n = varied from 40 to 616)	Systematic Review	School-based interventions targeting attitudes and stigma about mental illness.<br>The interventions varied in content and delivery methods: Nine were education-only and eight had indirect or direct contact with someone with lived experience.<br>The focus of the interventions was mental illness in 11 studies, schizophrenia in 3 and depression in 3.<br>Five studies investigated the impact of already established interventions.<br>The follow-up time ranged from immediately post intervention only, up to 12 months.	The duration ranged from one-off interventions lasting 30-120 min to multiple sessions over a period of up to 4 months	- A total of 13 of the instruments were designed for the intervention or study; 6 of these had poor (or untested) reliability<br>- Most measures were 'stigma' measures: attitudes, behavioural intentions and in one study an affect measure. No studies measured actual behaviour.<br>- All measurements were self-report Yes/No, True/False or Likert-style questionnaires, except for the Implicit Association Test (IAT)	- 17 studies were included<br>- 7 studies showed some statistically significant positive changes at follow-up; 4 studies report statistically significant positive results at immediate post-test only; 4 studies showed no significant changes at either posttest or follow-up<br>- There is no obvious pattern about what makes a successful intervention<br>- This review shows that there is no strong evidence to support that school-based interventions reduce stigma to mental illness in young people.	Unknown
Hart et al (2016)	Australia	Adolescents aged 14-17 years (n = 988)	Pilot study: an uncontrolled, pre-test/post-test design	*Teen Mental Health First Aid (teen MHFA)* is a classroom- based training program to increase mental health literacy, decrease stigmatising attitudes towards individuals with mental illness, and improve MHFA behaviours.<br>Education on mental health concept, mental health problems (types and impact), stigma, help seeking option, MHFA, mental health crises, teen MHFA action plan ("Look, Ask, Listen, Help, Your Friend"), helping a friend who is developing a mental health problem.<br>It was used didactic PowerPoint® presentation, video presentations, role-plays, group discussion, small group activities and a student booklet.<br>The program was facilitated by an accredited MHFA Instructor .	3 × 75 min classroom based training program across 5–8 school days	Students are assessed at baseline, and at 1 week, and at 3-month after the intervention - A survey questionnaire containing the following was developed by the authors:<br>- items adapted from the Australian National Survey of Youth Mental Health Literacy<br>- Open-ended questions about MHL (problem recognition and beliefs about help)<br>- 7 questions about personal stigma on a Likert scale<br>- Social Distance Scale adapted for young people<br>- Likert scale about confidence providing first aid<br>- Open-ended questions about MHFA intentions<br>- First Aid Experiences Questionnaire (modified)	- Improvement in MHL and in student's mental health<br>- Decrease in stigmatizing attitudes<br>- Increase in confidence in providing MHFA to a peer and in intentions to seek help<br><br>- *Teen MHFA program* is effective and feasible for delivery to secondary school students	Unknown

Table S1. Cont.

Author(s), Year	Country	Participants	Type of study	Programme / Interventions	Duration	Data collection instruments	Main outcomes	Barriers / facilitators
Hart et al (2018)	Australia	Adolescents aged 14-18 years (at 10th grade) (n = 1942) Intervention Group (tMHFA): n = 979 Control Group (PFA): n = 948	A cluster randomized crossover (CRXO) trial	*Teen Mental Health First Aid (tMHFA)* is a classroom-based training programme for students aged 15– 18 years to improve supportive behaviours towards peers, increase mental health literacy and reduce stigma.<br>Education on mental health concept, mental health problems (types and impact), stigma, help seeking option, MHFA, mental health crises, *teen MHFA* action plan, helping a friend who is developing a mental health problem.<br>It was used didactic PowerPoint® presentation, video presentations, role-plays, group discussion, small group activities and a student booklet.<br>The program was facilitated by an MHFA Instructor.	3 × 75 min classroom based training program across 5–8 school days	Students are assessed at baseline and at 1 week after the intervention:<br>- The survey, which was modified from previous national mental health literacy surveys with youth (Yap et al., 2012b) and evaluations of *tMHFA* (Hart et al., 2016), measured quality of MHFA intentions (helpful and harmful intentions, confidence in providing help), mental health literacy (problem recognition, beliefs about helpfulness of adult sources of help) and stigmatizing beliefs (social distance, weak-not sick, dangerous/ unpredictable and would not tell anyone)	- Compared to PFA (Physical First Aid), *tMHFA* resulted in significantly improved supportive first aid intentions and mental health literacy and significantly decreased stigmatizing attitudes among adolescents.<br>- *tMHFA* is an effective and feasible programme for increasing supportive first aid intentions and mental health literacy in adolescents in the short term	Unknown
Swartz et al (2017)	USA	Adolescents in 9th or 10th grade classes (n = 6679) Intervention Group (ADAP) n = 3681 Control Group n = 2998	Cluster Randomized Controlled Trial	Adolescent Depression Awareness Program (ADAP) is a universal school-based depression education program to increase depression literacy as the first step in encouraging depressed youths to seek treatment.<br>Education on knowledge about mood disorders (symptoms of depression, parallels between depression and other medical illnesses, potential consequence of depression – suicide, …) as well as attitudes about treatment.<br>It was used interactive lectures, videos, film assignments, homework, and group activities.<br>The program was delivered by school personnel - trained health education teachers.	Two 90-minute or three 45- to 60-minute class period (3 hours total given in 2–3 class periods)	Students are assessed at baseline, and at 6 weeks, and at 4-month after the intervention:<br>- The Adolescent Depression Knowledge Questionnaire (ADKQ)<br>- A version of the Reported and Intended Behavior Scale (RIBS)<br>- A modified version of the Child and Adolescent Services Assessment was used to access the ADAP's effect on the receipt of mental health services for depression and other conditions	- Increase in depression literacy in both boys and girls.<br>- Did not have effect on mental health stigma<br>- Adding ADAP to the standard high school health education curriculum implemented by health education teachers resulted in significantly higher levels of student depression literacy	**Facilitators:**<br>- Teachers implemented ADAP as part of the standard health education curriculum, which does not compete for academic time and encourages sustainability

Table S1. Cont.

Author(s), Year	Country	Participants	Type of study	Programme / Interventions	Duration	Data collection instruments	Main outcomes	Barriers / facilitators
Schilling et al (2016)	USA	Adolescents in 9th grade (n = 1052)	Pre-test post-test randomized control design	*SOS (Signs of Suicide)* is a universal school-based suicide prevention program.<br>Education on warning signs of suicide risk, on differences between major depression disorder and stress or emotional upset; and training to seek-help for themselves and/or for a friend.<br>It was used presentations, group discussions and videos (included dramatizations and also interviews with real people).<br>The program was delivered by school counseling and social work staff (after a 1-day training).	Unknown	Students are assessed at baseline (pre-test) and at 3-month after the intervention (post-test):<br>- Questions/items adapted from the Youth Risk Behavior Survey (YRBS)	- Decrease in self-reported suicide attempts in the 3 months following the program<br>- Increase in knowledge about depression and/or suicidal thoughts<br>- Increase favorable attitudes toward help-seeking for themselves or for friends<br>- No changes on suicidal ideation	Unknown
Kutcher, Wei & Morgan (2015)	Canada	Students in 9th grade (n = 175)	Secondary analysis	*The Guide* (The Mental Health and High School Curriculum) is a web-based Mental Health Literacy (MHL) curriculum resource for use in junior high and secondary schools.<br>Education on stigma of mental illness, mental health and wellness, mental health disorders, experiences of mental illness, seeking help and finding support, and on the importance of positive mental health.<br>It was used written materials, animated videos, PowerPoint® presentations and classroom activities.<br>The intervention was delivered by the students' usual teachers.	Total of 10/12 hours of class time (6 modules)	Students were assessed pre- and post-intervention, and at 2-month follow-up:<br>- Knowledge and attitude Survey (developed by authors)	- Improvement in knowledge (post-intervention and at 2-month follow-up)<br>- Improvement in attitudes toward mental disorders/illness post- intervention and at 2-month follow-up)	**Facilitators:**<br>- Improves both teacher and student MHL with the same activity<br>- It does not require additional investment and it is not dependent of external mental health experts because it is integrated into existing health curriculum

Table S1. Cont.

Author(s), Year	Country	Participants	Type of study	Programme / Interventions	Duration	Data collection instruments	Main outcomes	Barriers / facilitators
Milin et al (2016)	Canada	Students in 11th and 12th grade (n=534)<br>Intervention Group (The Guide)<br>n = 362<br>Control Group (teaching As Usual)<br>n = 172	Randomized Controlled Trial	*The Mental Health and High School Curriculum Guide (The Curriculum Guide)* was required to be integrated within the grade 11 or 12 Ontario Provincial Ministry of Education Healthy Living course.<br>Education on stigma of mental illness, mental health and wellness, mental health disorders, experiences of mental illness, seeking help and finding support, and on the importance of positive mental health.<br>It was used classroom activities, core, and supplementary resources, such as digital stories and video interview of youth with mental illness.<br>The intervention was delivered by the students' teachers of the Healthy Living course.	6 hours of class time (6 modules)	Students were assessed pre- and post-intervention:<br>- Knowledge and attitude survey (developed by the original authors of The Guide)	- Students who received The Curriculum Guide showed significant improvements in mental health knowledge and a reduction in stigma compared to those receiving TAU<br>- It was found that improvement in mental health knowledge predicted a corresponding improvement in attitudes toward mental illness/reduction in stigma	**Facilitators:**<br>- Improves both teacher and student MHL with the same activity<br>- It does not require additional investment and it is not dependent of external mental health experts because it is integrated into existing health curriculum
Chisholm et al (2016)	UK	Students in 8th grade, aged 12–13 years (n = 657)	Cluster Randomized Controlled Trial	The *SchoolSpace* is educational programme combined with intergroup contact to reduce the stigma of mental illness and increase the mental health literacy.<br>Education on stigma, mental health problems and on mental illness. Plus, a Contact Session with a young person who experienced a mental illness.<br>It was used presentations, drama workshop and direct contact with a mentally ill young person.<br>The intervention was delivered by mental health professional staff and contact volunteers.	1 day	Students were assessed at the baseline and at 2-week follow-up:<br>- Reported and Intended Behaviour Scale (RIBS)<br>- Mental Health Knowledge Schedule (MAKS)<br>- Strengths and Difficulties Questionnaire (SDQ)<br>- a 15-item version of the Resilience Scale<br>- Attitudes to help-seeking were assessed by responses on a seven-point scale to one question<br>- Acceptability of the intervention was assessed in one school using two short group interviews	- Attitudinal stigma improved in both conditions with no significant effect of condition.<br>- The education intervention appeared to be successful in reducing stigma, promoting mental health knowledge, and increasing mental health literacy, as well as improving emotional well-being, resilience and help seeking attitudes.<br>- Intergroup contact was not seen to add value.	Unknown

Table S1. Cont.

Author(s), Year	Country	Participants	Type of study	Programme / Interventions	Duration	Data collection instruments	Main outcomes	Barriers / facilitators
Ojio et al (2018)	Japan	Students in 5th and 6th grade (n = 662)	An uncontrolled, pre-test/post-test design	*The Short MHL Program for Pre-Teens (SMHLP for pre-teens)* is a concise teacher-led program for mental health literacy (MHL).<br>Education on mental health problems (prevalence, risk factors, …) and on help-seeking.<br>It was used a 10-minute animated film.<br>The program was delivered by the school teacher's during the health education classes.	45-min session	Students were assessed at the baseline, at post-intervention and at 3-month follow-up:<br>- Questionnaire (developed by the authors) that evaluate knowledge about mental health/illnesses; Recognition of mental health state of a character in a vignette; recognition of the necessity to seek help; intention to seek help (self); intention to help (peers)	- Improvement in knowledge about mental health/illness, in recognition of a mental health state, and in intention to help (peers)	**Facilitators:**<br>- Program being concise<br>- Program delivered by internal staff
Ojio et al (2015)	Japan	Students in 9th grade aged 14-15 years (n = 102)	An uncontrolled, pre-test/post-test design	A concise, school-staff-led MHL program for adolescents to promote mental health literacy (MHL).<br>Education on mental illness (prevalence, onset age, risk factors, treatability, recovery, symptoms in adolescence); on sources of help; on methods of psychiatric diagnosis and clinical examinations for it.<br>It was used standard instructions, animations and group discussions.<br>The program was delivered by the school teachers during the health education classes.	Two 50-min sessions once a week over a 2-week period	Students were assessed at the baseline, at post-intervention and at 3-month follow-up:<br>- Questionnaire (developed by the authors) that evaluate knowledge about mental health/illnesses; Recognition of mental health problems and selection of desirable behavior; intention of helping peer with mental health problems	- Improvement in knowledge about mental health/illness and about their treatment; in recognition of a mental health problems and correct selection of desirable solution; and in intention of helping peers with mental health problems.	**Facilitators:**<br>- Program delivered by the usual school teachers
Skre et al (2013)	Norway	Adolescents in secondary schools (13–15 years) (n=1070)<br>Intervention Group: n = 520<br>Control Group: n = 550	Non-randomized cluster controlled trial	*Mental health for everyone* is a universal education programme to promote the mental health literacy.<br>Education on prevention of mental health disease; on attitudes and prejudices against mental health problems and the mentally ill; on openness and confidence about mental health issues; and on mental health services available to help.<br>It was used individual tasks, group tasks and plenary sessions, and illustrating video material.<br>The program was delivered by the school teachers with a small direct/indirect help of the authors of the programme when necessary.	3 days	Students were assessed at the baseline and at 3-month follow-up:<br>- A 66-item questionnaire, of which 7 questions were open ended, was employed (developed by the authors):<br>a) Demographic variables<br>b) Measurement of mental health literacy: Symptom profile recognition; Prejudiced beliefs; Knowledge about where to seek help for mental health problems.	- Improvement in mental health literacy in terms of symptom profile identification, prejudiced beliefs, and knowledge about where to seek help	**Facilitators:**<br>- Program delivered by the usual school teachers<br>- Short programme

Table S1. Cont.

Author(s), Year	Country	Participants	Type of study	Programme / Interventions	Duration	Data collection instruments	Main outcomes	Barriers / facilitators
Gonçalves et al (2015)	Portugal	Adolescents in 7th, 8th and 9th grades (n = 207) Intervention Group n = 115 Control Group n = 92	Pilot study: Cluster Randomized Controlled Trial	Is a video-based mental health de-stigmatization intervention about the experience of a female adolescent that sought professional help. It was used video watching and group discussion. It is unclear who delivered the intervention.	10-min	Adolescents were accessed at pre, post and 1-month follow-up: - Self Stigma of Seeking Help Scale (SSOSH) - Social Stigma for Receiving Psychological Help Scale (SSRPH) - the Attribution Questionnaire-Children form (AQ-8-C)	- Decrease in self-stigma for seeking help, in social stigma for seeking help and in attribution, at the post-time period but not at 1-month follow-up.	**Facilitators:** - Quick and inexpensive intervention
Salerno (2016)	USA	Students enrolled in US K-12 schools, from 5th to 12th grade (n = varied from 30 to 5949)	Systematic Review	K-12 school-based mental health awareness interventions in the United States to improve mental health/illness knowledge, improve attitudes toward mental health or illness, and increase help-seeking. The interventions varied in content and delivery methods: primarily instructor-led traditional mental health education curriculums, some included one-time presentations or videos. Interventions were delivered by a faculty adviser, counselor, teacher, nurse, researcher, clinician, mental health professional, staff member, or consumer.	The duration ranged from 1-time educational presentation to multiple sessions	Unknown	- 15 studies were included - Only 3 of the 15 studies measured all 3 outcomes: knowledge, attitudes, and help-seeking. - All studies measuring knowledge demonstrated improvements. - Most of the studies measuring attitudes and help-seeking also demonstrated improvements.	**Barriers:** - coordinating with the various stakeholders in schools, disruption of school curriculums, parental consent, and lack of incentives.
Martínez-Zambrano et al (2013)	Spain	Secondary school students, aged 14-16 years (n = 62)	An uncontrolled, pre-test/post-test design	The program consisted in providing information and contact with users of mental health in order to reduce social stigma in the school environment. Education on mental illness (concept, causes, erroneous ideas, hospital and community resources, feelings and social network of mentally ill), and contact with people suffering mental disorders and with mental health professionals. It was used audiovisual techniques (videos), individual tasks and open dialogue/discussion. The program was delivered by 2 psychologists, 1 social worker and 1 nurse during the class.	2 sessions taking place during 2 weeks	Students were assessed before and after the intervention: - Opinions on Mental Illness (OMI) questionnaire	- In all the subscales of the instrument the students improved their perception of mental disorders, reducing their levels of stigma - The intervention was effective in reducing social stigma among young adolescents	Unknown
